# Ligation-Mediated Polymerase Chain Reaction Detection of 8-Oxo-7,8-Dihydro-2′-Deoxyguanosine and 5-Hydroxycytosine at the Codon 176 of the p53 Gene of Hepatitis C-Associated Hepatocellular Carcinoma Patients

**DOI:** 10.3390/ijms21186753

**Published:** 2020-09-15

**Authors:** Andrea Galli, Armelle Munnia, Filippo Cellai, Mirko Tarocchi, Elisabetta Ceni, Frederik Jan van Schooten, Roger Godschalk, Roger W. Giese, Marco Peluso

**Affiliations:** 1 Department of Experimental and Clinical Biomedical Sciences, University of Florence, 50139 Florence, Italy; a.galli@dfc.unifi.it (A.G.); m.tarocchi@dfc.unifi.it (M.T.); e.ceni@dfc.unifi.it (E.C.); 2 Research Branch, Regional Cancer Prevention Laboratory, ISPRO-Study, Prevention and Oncology Network Institute, 50139 Florence, Italy; a.munnia@ispro.toscana.it (A.M.); f.cellai@ispro.toscana.it (F.C.); 3 Department of Pharmacology & Toxicology, Maastricht University, 6229 ER Maastricht, The Netherlands; f.vanschooten@maastrichtuniversity.nl (F.J.v.S.); r.godschalk@maastrichtuniversity.nl (R.G.); 4 Bouve College of Health Sciences, Barnett Institute, Northeastern University, Boston, MA 02115, USA; r.giese@northeastern.edu

**Keywords:** HCC, HCV, p53, 8-oxodG, M_1_dG

## Abstract

Molecular mechanisms underlying Hepatitis C virus (HCV)-associated hepatocellular carcinoma (HCC) pathogenesis are still unclear. Therefore, we analyzed the levels of 8-oxo-7,8-dihydro-2′-deoxyguanosine (8-oxodG) and other oxidative lesions at codon 176 of the p53 gene, as well as the generation of 3-(2-deoxy-β-d-erythro-pentafuranosyl)pyrimido[1,2-α]purin-10(3H)-one deoxyguanosine (M_1_dG), in a cohort of HCV-related HCC patients from Italy. Detection of 8-oxodG and 5-hydroxycytosine (5-OHC) was performed by ligation mediated-polymerase chain reaction assay, whereas the levels of M_1_dG were measured by chromatography and mass-spectrometry. Results indicated a significant 130% excess of 8-oxodG at –TGC– position of p53 codon 176 in HCV-HCC cases as compared to controls, after correction for age and gender, whereas a not significant increment of 5-OHC at –TGC– position was found. Then, regression models showed an 87% significant excess of M_1_dG in HCV-HCC cases relative to controls. Our study provides evidence that increased adduct binding does not occur randomly on the sequence of the p53 gene but at specific sequence context in HCV-HCC patients. By-products of lipid peroxidation could also yield a role in HCV-HCC development. Results emphasize the importance of active oxygen species in inducing nucleotide lesions at a p53 mutational hotspot in HCV-HCC patients living in geographical areas without dietary exposure to aflatoxin B_1_.

## 1. Introduction

Hepatocellular carcinoma (HCC) is the most common primary liver malignancy and the second leading cause of cancer death [1]. Notably, HCC is predominant in sub-Saharan Africa and Eastern Asia, geographic areas where the major risk factors are hepatitis B virus (HBV) infection and dietary exposure to aflatoxin B_1_ (AFB_1_) a fungal mycotoxin, which contaminates foods [1]. Conversely, the main risk factors for HCC development in European Union is the chronic infection with hepatitis C virus (HCV) [2]. Especially, the highest incidence of HCC has been reported in Italy, with an age standardized rate of 7.1 cases per 100,000 persons [3]. Although recent advances in liver cancer research have increased our knowledge of HCC pathogenesis, the molecular mechanisms underlying HCV-associated HCC pathogenesis are still unclear. A recent study highlighted the importance of *tumour suppressor P53* (*TP53*) in HCC pathogenesis [4]. Whole-exome and next generation sequencing data showed that the frequency of p53 mutations is ranging from 28 to 54% in liver tumors from the Cancer Genome Atlas and a Chinese clinical dataset [5]. However, somatic p53 mutations have variable frequencies in different geographic areas, depending on liver disease etiology and environmental parameters [6]. For instance, in Asia and Africa, hepatitis B virus infection and dietary exposure to AFB_1_ has been associated with G:C to T:A transversions at the third base in codon 249 of the p53 gene causing R249S substitution. Outside the R249S mutation, G:C to T:A transversions at codon 176 can be over-represented in 28–39% of HCV-related HCCs [2]. In this case, a direct mutagenic effect of HCV proteins, that deregulate host cell cycle checkpoints and induce virus and immune-mediated oxidative stress [7], leading to somatic mutations in hepatic cells has been suggested [8]. Specially, reactive oxygen species (ROS) are chemicals able to damage DNA leading to oxidative DNA damage [9], including 7-hydro-8-oxo-2′-deoxyguanosine (8-oxodG) [9]. 8-oxodG, if not fully repaired, can induce G:C to T:A transversions and G:C to A:T transitions, including in liver cancer [10]. ROS can also induce lipid peroxidation (LPO) of cellular membranes [9]. Particularly, malondialdehyde (MDA) is a highly reactive aldehyde able of reacting with DNA forming 3-(2-deoxy-β-d-erythro-pentafuranosyl)pyrimido[1,2-α]purin-10(3H)-one deoxyguanosine (M_1_dG) [11]. M_1_dG is a kind of adduct which is able to block cell replication and to induce base pair and frameshift mutations [12]. M_1_dG has then been correlated with epigenetic alterations in the Long Interspersed Nuclear Element-1 repeated sequences and in the promoter region of the inflammatory cytokine *interleukin-6* gene [13,14]. Moreover, oxidative DNA damage has been linked to cancer development and tumor progression [15,16,17,18]. Recently, we examined the levels of 8-oxodG on p53 sequence by ligation-mediated polymerase chain reaction (LM-PCR) assay [19]. In that study, significant increments of 8-oxodG at the mutational hotspot codons 163 and 175 were found in breast cancer women relative to controls, indicating a causal relationship between adducts and breast cancer.

Therefore, we conducted an hospital-based study aimed at comparing the presence of 8-oxodG and other base lesions at codon 176 of the exon 5 of the p53 gene and the levels of M_1_dG in the genomic DNA in the hepatocytes of HCV-HCC patients in respect to controls in Italy, a country with a high HCC incidence [3]. The generation of oxidative DNA lesions was also examined in liver hepatocellular (HepG2) cells, which were exposed to a ROS-generating system [20]. The analysis of 8-oxodG and 5-hydroxycytosine (5-OHC) at the sequence level of the p53 gene was performed by LM-PCR [19], whereas the detection of M_1_dG by chromatography and mass spectrometry [16,21]. Our purpose was to identify biomarkers of hepatocarcinogenesis that could be used for the early detection of susceptible individuals in cohorts of patients with chronic HCV infections and other liver diseases.

## 2. Results

### 2.1. Demographic Variables

The present liver cancer case-control study involved 53 volunteers. There were 31 HCC cases who were HCV infected, mean age 58.7 ± 6.2 years (standard deviation, SD) and 22 controls, with average age 59.9 ± 6.1 (SD). Controls were 19 patients with secondary liver metastatic cancer and 3 obese individuals with steatosis.

### 2.2. Measurement of M_1_dG Standard

The levels of M_1_dG per 10^6^ nucleotides expressed, such as relative adduct labeling (RAL), were 5.0 ± 0.6 (Standard Error, SE) in the malondialdehyde-treated calf-thymus DNA by ^32^P-DNA post-labeling [16]. The formation of M_1_dG in the treated calf-thymus DNA sample was confirmed by the matrix-assisted laser desorption/ionization time-of-flight mass spectrometry [21]. Relative to the accurate masses that were found in the spectrum from one spot, where the exact mass was of (581.166) by the M nomenclature of Goda and Marnett [22]: M_1_dG (581.166). A calibration curve was then set up by diluting the reference standard with untreated calf-thymus DNA and measuring the decreasing adduct levels, r-squared = 0.99.

### 2.3. Detection of 8-oxodG, 5-OHC, and M_1_dG in HCV-Associated HCC Patients and HepG2 Cells

**Figure 1:**
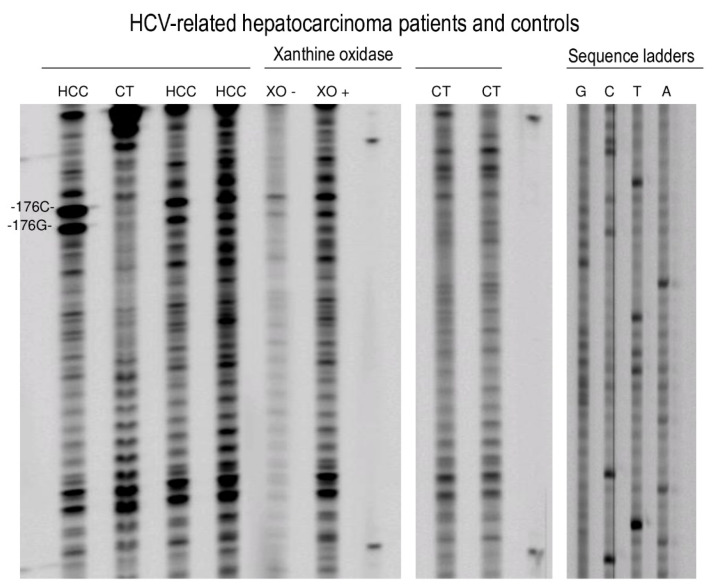
Sequencing gels showing the intensities of oxidative adduct-induced formamidopyrimidine DNA glycosylase (Fpg) and endonuclease III (EndoIII) incisions at the codon 176 along the exon 5 sequence of the p53 gene of hepatitis C virus (HCV)-associated hepatocellular carcinoma (HCC) patients and controls (Lanes 1–4, 8, and 9 from the left side), as well as human hepatocytes treated with 0.2 mM xanthine plus 5.0 mU xanthine oxidase (XO)-treated (Lanes 5 and 6). Autoradiograms: Lanes: 1, 3, and 4, HCV-HCC patients; Lanes 2, 8, and 9, controls; Lane 5, XO treatment; Line 6, no XO treatment; Lanes 7 and 10 molecular weight; Lanes 11–14, sequencing ladders. Heavy modified –G– and –C– positions at codon 176 are labeled.

**Figure 2:**
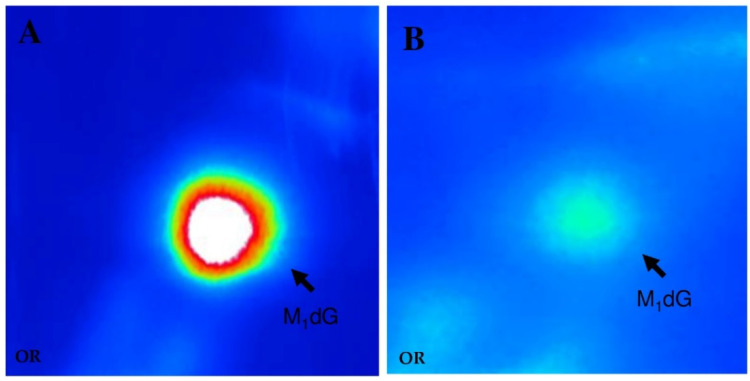
Characteristic chromatographic pattern of 3-(2-deoxy-β-d-erythro-pentafuranosyl)pyrimido[1,2-α]purin-10(3H)-one deoxyguanosine (M_1_dG) detected in the calf-thymus DNA exposed to 10 mM MDA (**A**) and in the hepatocytes of hepatitis C virus hepatocellular carcinoma patients (**B**).

Representative sequencing gels in Figure 1 show that stronger adduct binding occurs at the codon 176 along the exon 5 sequence of the p53 gene of the hepatocytes of HCV-HCC patients as compared to controls. Relative to the control group, sequencing gels indicated that there were no significant differences in band intensity between subjects with secondary liver metastatic cancer and obese individuals with steatosis. Table 1 reports the mean intensities, expressed in Relative Intensity (RI) estimates, of 8-oxodG and 5-OHC-induced formamidopyrimidine DNA glycosylase (Fpg) and endonuclease III (EndoIII) incisions were 0.95 ± 0.27 (SE) and 0.61 ± 0.40 (SE) in HCV-associated HCC cases, whereas 0.29 ± 0.01 (SE) and 0.28 ± 0.06 (SE) were observed in controls, respectively. Results of multivariate analysis in Table 2 showed that a significant 130% excess of 8-oxodG was present at codon 176 (–TGC–) of HCV-related HCC cases as compared to controls, after correction for age and gender (*p* < 0.0001). Conversely, the increment of 5-OHC at codon 176 (–TGC–) did not reach the statistical significance (*p* = 0.115). Chromatographic findings showed a typical M_1_dG spot pattern which was detected in the standard (Figure 2A) as well as in the liver cancer patients (Figure 2B). Results in Table 1 show that the greater level of M_1_dG, expressed in RAL × 10^8^, was found in HCV-associated HCC cases relative to controls: There were 3.25 × 10^8^ ± 0.41 (SE) and 1.67 × 10^8^ ± 0.18 (SE) in HCC patients and controls, respectively. Multivariate analyses using log-normal regression models have also shown that an 87% excess of M_1_dG occurred in HCV-related HCC cases relative to controls, after adjusting for age and gender. When statistical analyses were repeated after excluding the 3 obese subjects with steatosis, a disease often characterized by high ROS and LPO [23], no significant changes were found in the results.

Results in Figure 1 show also that HepG2 cells exposed to a ROS generating system present heavier bands in the sequencing gel relative to untreated cells. Elevated levels of 8-oxodG and 5-OHC were found in the non-transcribed strand at codon 176 (–TGC–) on *TP53* of ROS-treated HepG2 cells, as reported in Table 1. The mean intensities, expressed in RI estimates, of 8-oxodG and 5-OHC- induced Fpg and EndoIII incisions, were 0.44 ± 0.04 (SE) and 0.34 ± 0.03 (SE) in treated cells, respectively, whereas 0.22 ± 0.02 (SE) and 0.20 ± 0.01 (SE) values were found in control cells, respectively. Then, ^32^P-postlabeling findings show that the formation of M_1_dG was significantly higher within treated HepG2 cells relative to untreated cells (*p* < 0001). The levels of M_1_dG, expressed in RAL × 10^8^, were 3.2 ± 0.3 (SE) and 0.35 ± 0.2 (SE), in treated and control HepG2 cells, respectively.

Next, in order to investigate the biological consequence of 8-oxodG and 5-OHC production, we have examined the frequencies of p53 mutations in HCV-associated HCC tumors in the International Agency for Research on Cancer *TP53* database [24]. In Table 3, there are the levels of susceptible lesions, as well as the mutational signatures and the rates of p53 mutations, detected in HCV-related HCCs. Our findings show that the generation of oxidative nucleotide lesions occurs in sites where the percentages of transition and transversion mutations are ranging from 8% to 42%. This p53 mutational spectrum is also expected in response to ROS exposure, since the type of mutations induced by 8-oxodG and 5-OHC consist mostly in G:C > T:A and G:C > A:T [25].

## 3. Discussion

Inflammation has an important role in the occurrence and development of cancer [26,27], including HCC, that arises almost exclusively in the setting of chronic inflammation [28]. Several inflammatory cells and mediators are commonly present in stromal cells and tumor microenvironments [27]. Some of them, such as neutrophils and macrophages, release ROS that promote DNA damage [29]. An interplay between ROS and p53, with an increasing antioxidant role of the wild-type in the regulation of inflammation, has been demonstrated [30]. In addition to be a guardian of the genome, p53 is a regulator of intracellular metabolism that acts as a transcriptional regulator capable of inhibiting oxidative stress in inflammatory microenvironments [31]. Through increasing the expression of antioxidant genes, such as *glutathione peroxidase* and *aldehyde dehydrogenase 4*, p53 can prevent excessive ROS production that could compromise genome integrity [32,33]. In contrast with the wild-type, mutant p53 proteins sustain ROS formation and promote tumor progression by regulating Nrf2 and PGC-1α target genes [34]. 

The most remarkable result of the present study is the high frequencies of a reproducible characteristic pattern of oxidative base lesions along the exon 5 sequence of the p53 gene among HCV-related HCC patients in respect to controls. Specifically, we have detected by LM-PCR a strong and selective formation of 8-oxodG and 5-OHC at 2nd and 3th nucleotides of the codon 176 (–TGC–), a site where somatic p53 mutations can be over-represented according to the IARC *TP53* database [24]. Of note, guanine residue was the major site of oxidative nucleotide lesions (95% Confidence Interval (C.I.), 1.57–3.48), whereas a not significant increment was determined at cytosine position (95% C.I. 0.71–20.3). In sum, selective oxidative DNA binding rather than phenotypic selection appears to be responsible of a typical mutational pattern in HCV-associated HCC [24]. Notably, the site of this mutational hotspot is different from that generally found at the codon 249 in exon 7 on p53 in HBV-infected HCC patients who consume food contaminated with AFB_1_ [24]. Moreover, the absence of mutations at the codon 249 on the p53 gene of HCC patients has been recently reported in Italy [6], a geographic area where there is no dietary exposure to AFB_1_ and where the general prevalence of HBV infection is lower than 1%.

Guanine and cytosine appear to be two nucleotides that are preferentially sites of oxidative DNA binding at the codon 176 along the sequence of the exon 5. This has a special value since greater generations of 8-oxodG and 5-OHC at base level along specific sites of the p53 sequence can reflect higher probability of nonrandom somatic p53 mutations at the same positions on hepatocytes during cell division and replication. Furthermore, our in vitro results are in line with previous investigations [19,35], which examined the generation of oxidative base lesions along the sequence of the p53 gene, after ROS treatment in human adenocarcinoma, epithelial lung, and MDA-MB23 estrogen receptor negative (ER−) breast cells. The complete correspondence between the site of preferential adduct binding detected in HCV-HCC cases with those observed hepatocytes in vitro treated with a ROS-generating system, e.g., the xanthine plus xanthine oxidase system [20], as well as with an over-represented mutational profile, e.g., the codon 176, in HCV-HCC tumors [24], strongly indicates that oxidative stress and ROS potentially produced during viral infection and inflammatory processes can be behind the high rates of G:C to T:A transversions and G:C to A:T transitions in HCV-HCC tumors [24]. Furthermore, multivariate regression analysis demonstrated that the frequency of M_1_dG was significantly enhanced in HCV-HCC patients compared to controls, with a Mean Ratio (MR) value of 1.87 (95% C.I. 1.27–2.77), supporting the role of HCV in exploiting the host lipid machinery not only to replicate and spread but also to produce highly reactive aldehydes [36]. Nonetheless, considering the long latency period of HCV-positive HCC before clinical symptoms, we argued that a single mutation should not be sufficient for cancer development. Additional hits, perhaps in a particular order, in other cancer drivers, such as in the oncogene *Catenin beta-1*, and in the promoter region of *telomerase reverse transcriptase* [37,38,39] are probably required to confer differential selective advantages.

Currently, there is growing evidence that HCV-induced oxidative stress significantly plays a critical role in hepatic fibrogenesis and carcinogenesis [40]. Our results support the role of ROS production and the reduction of free radical scavenging capacity in hepatocarcinogenesis in keeping with previous investigations, where the frequencies of oxidative damage have been analyzed [41,42]. For instance, in the study of Shawki [41], the levels of oxidative peripheral blood DNA damage, as measured by comet assay, have been found to be significantly higher in both HCV-HCC cases in respect to controls. Elevated amounts of hepatic 8-oxodG among HCV-positive patients have been associated to HCC development [43], as well as to postoperative recurrence [44] and shorter survival [45]. HCV core proteins could act as a tumor promoter by facilitating the mutational potential of 8-oxodG, thus connecting an HCV infection with hepatocarcinogenesis. Increased generation of oxidative DNA damage in infected hepatocytes can be caused by several mechanisms: for an example, HCV expressed proteins can induce oxidative stress in hepatocytes through impairing directly the mitochondrial respiratory chain through a ROS overproduction, that alter both mitochondria’s structure and hepatocyte function [46]. Oxidative stress can arise from the dysregulation of calcium signaling in the endoplasmic reticulum/mitochondria junctions [46]. 3*β*-hydroxysterol Δ24-reductase can also play a critical role in hepatocarcinogenesis through the suppression of nuclear p53 activity by blocking its acetylation and increasing its interaction with the E3 ubiquitin protein ligase cytoplasm protein leading to its degradation [46]. Additional support to HCV-induced oxidative stress hypothesis is provided from studies on hemochromatosis [2], where a link between iron overload, p53 mutations and hepatocarcinogenesis has been suggested in patients with hemochromatosis.

Molecular epidemiology of HCCs has shown a significant variability between geographic areas depending on several risk factors, including those prevailing in each region [2]. The best characterized alterations are those occurring at the third base in codon 249 of the p53 gene causing R249S mutation in patients with HBV infection combined with the dietary consumption of food contaminated by AFB_1_ [36]. Conversely, the HCV-induced mutator phenotype has been generally associated to the ability of this virus to cause double strand DNA breaks and to activate error-prone DNA polymerases and cytidine deaminase [47]. Theoretically, colocalization of nucleotide lesions and mutational hotspot could be used for causality inference. Our data demonstrated that there is a direct etiological link between oxidative nucleotide lesions, caused by a defined chemical carcinogen (endogenous ROS), with HCV-HCC development. These considerations are in keeping with those of a recent study of Ceppi et al. [48] that demonstrated, by a meta-analytic approach, that the induction of DNA adducts is not simply related to carcinogen exposure but is a cause of chemical-induced cancer. High base lesions at the codon 176 of the exon 5 of the p53 gene could have various deleterious consequences, such as impaired replication, enhanced mutagenesis, and decreased binding affinity of transcription factors, which, in turn, could cause an inappropriate reactivation of silenced oncogenes. However, some caution should be used, when trying to assess liver cancer risk inherent to p53 mutations potentially induced by 8-oxodG since further mutational hits in other cancer driver genes and additional mechanisms could be required to confer differential selective advantages and ultimately lead to liver cancer. Additionally, cell transformation does not occur with every interaction between carcinogens and DNA; hence, it is likely that only a relatively small proportion of such nucleotide lesions can induce somatic p53 mutations.

Several parameters can influence the pattern of base lesions and their biological impact on hepatocarcinogenesis. For example, chromatin structure can be involved in determining the site-selectivity of genotoxic species. The difference in base lesions also can reflect the fact that some sites are repaired more readily than others. Other factors, such as DNA sequence context or cytosine methylation patterns, can be involved in shaping the oxidative adduct profile on *TP53* [49]. Along the sequence of exon 5 on the p53 gene, various CpG sites were identified as hotspot of adduction, but their methylation status is unknown. In addition, HCC is not a unique category, because some tumors are well differentiated, whereas others are little differentiated with an aggressive evolution. The latter can generally belong to the fraction with deleterious p53 mutations, that shows higher rates of recurrence and shorter disease-free survival [50]. Since HCC presents significant heterogeneity among tumors, it would have been also interesting to integrate adduct data with clinical parameters to form a classification scheme. Nonetheless, we could not attempt to classify primary liver tumor by molecular subgroups because of limited sample size.

In conclusion, our study provided evidence that premutagenic lesions on the p53 gene can contribute to HCV-associated hepatocarcinogenesis. Findings further emphasize the importance of ROS in inducing nucleotide lesions at a p53 mutational hotspot in HCV-HCC patients who were resident in geographical areas where HBV prevalence is low and where there is no dietary exposure to aflatoxin B_1_. The analysis of 8-oxodG and 5-OHC at the p53 sequence level could be used for the early detection of susceptible patients requiring a more intense clinical surveillance in cohorts of subjects with chronic HCV infections and other liver diseases.

## 4. Material and Methods

### 4.1. Cell Culture, ROS Treatment, and DNA Isolation

HepG2 cells (5 × 10^6^, ATCC, Manassas, VA, USA) were grown in RPMI 1640 medium with 10% fetal bovine serum (Sigma, St Louis, MO, USA) at 37 °C with 5% CO_2_ on plates 150 mm in diameter. HepG2 cells were treated at 30–40% confluence with 0.2 mM xanthine (Sigma, St Louis, MO, USA) plus 5.0 mU xanthine oxidase (Sigma, St Louis, MO, USA) to generate ROS. DNA was extracted and purified from cell pellet or liver specimens with organic solvents after treatments with RNase A and T_1_ and proteinase K (Sigma, St Louis, MO, USA) [51]. DNA levels and purity were analyzed using a Beckman DU 800 spectrophometer (South Kraemer, CA, USA). Coded DNA samples were stored at −80 °C.

### 4.2. Study Participants

HCV-HCC cases and controls were recruited from patients undergoing surgery for HCC or for colorectal cancer or undergoing clinical investigation for diagnostic purpose at General Hospital. After informed consent, liver specimens were collected during surgery, snap-frozen, and stored at −80 °C until DNA extraction and purification. Histopathological HCC diagnosis was obtained from the Pathological yard. The study was approved by the Institutional Review Board of the General Hospital (18 March 2013, N.17468) and performed in accordance with the ethical standards of the Declaration of Helsinki.

### 4.3. Ligation Mediated-PCR Assay

Mapping of oxidative base lesions in the nontranscribed strand at the codon 176 along the exon 5 sequence of the p53 gene was performed by LM-PCR [19], an assay measuring nucleotide lesions via conversion into DNA single-strand breaks. The enzymatic cleavage of DNA was performed by treatment with formamidopyrimidine DNA glycosylase (Fpg, Merck, Kenilworth, NJ, USA) and endonuclease III (EndoIII, BioLabs, Ipswich, MA, USA), two *Escherichia coli* repair enzymes that recognize various kind of oxidative nucleotide lesions [52]. Briefly, DNA samples (2 µg) were incubated with 0.4 U bacterial Fpg (0.2 U/µL) and 200 U bacterial Endo III (100 U/µL) for 60 min. Fragments with ligatable sequences were amplified with 16.0 pmol of a primer with the following sequence 5′-GGCAACCAGCCCTGTCG and a calculated *T_m_* of 56 °C, as well as 3 U *Thermococcus litoralis* exo-DNA polymerase (2 U/µL, BioLabs, Ipswich, MA, USA), 0.6 mM dNTPs (Sigma, St Louis, MO, USA), and 0.5 mM MgSO_4_ (Merck, Kenilworth, NJ, USA). The following program was used: denaturation at 95 °C for 2 min, annealing at 54 °C for 2 min, and elongation at 72 °C for 2 min (1 cycle), followed by 20 cycles of 1 min at 95 °C, 2 min at 54 °C, and 3 min at 72 °C. The blunt ended fragments with 5′-phosphate termini were incubated overnight with Ligase Buffer, 0.05 mg/mL bovine serum albumin (Sigma, St Louis, MO, USA), 60 pmol of the asymmetric double-stranded unphosphorylated linker (α-beta), and 90 U T4 ligase (400 U/µL, BioLabs, Ipswich, MA, USA) at 17 °C for having a common sequence at all 5′-ends. After this step, linker-ligated fragments were evaporated to dryness and resuspended in water. Then, amplification and labeling reactions were done with 12.0 pmol of a IRDye^®^ 700/800 fluorescence-labeled primer (LICOR, Lincoln, NE, USA) with the following sequence 5′-TCTCTCCAGCCCCAGCTGCTCAC and a calculated *T_m_* of 61 °C, as well as 12.0 pmol of the LP25 universal linker primer with 1 U of Vent (exo-) DNA polymerase (2 U/μL, BioLabs, Ipswich, MA, USA), 0.3 mM dNTPs, and 2.5 mM MgSO_4_ (Merck, Kenilworth, NJ, USA). The following PCR conditions were used: denaturation at 95 °C for 2 min (1 cycle), followed by 10 cycles of 45 s at 95 °C, 3 min at 59 °C, and 3 min at 72 °C. The IRDye^®^ 700/800 fluorescence-labeled products (2 μL) were denaturated with formamide loading buffer at 95 °C for 5 min. Denaturated labeled products were subjected to electrophoresis on a polyacrylamide sequencing gel using a LICOR 4300 (Lincoln, NE, USA) DNA analyzer at 65 °C for 7 h. DNA single-strand break quantification was obtained by ImageQuant (GE Health Care, Amersham, UK). Base lesions were expressed as relative band intensity (RI) = I_j_/I_max_, where I_j_ is the intensity in pixels of each band after background subtraction and I_max_ is the mean intensity in pixels of the highest intensity bands [35]. RI values were corrected across experiments based on the recovery of internal standard. The exact position of each nucleotide was determined by including labeled DNA fragments obtained by sequencing of the region of interest according to Ruano and Kidd [53] and appropriate IRDye^®^ 700/800 sizing standard markers in sequencing gels.

### 4.4. Preparation of Reference Adduct Standard

Calf-thymus DNA (Sigma, St Louis, MO, USA) was exposed to 10 mM MDA (ICN Biomedicals, Costa Mesa, CA, USA), as previously described [20]. MDA-treated calf-thymus DNA was diluted with untreated DNA to obtain lower amounts of the reference adduct standard to generate a calibration curve. 

### 4.5. Mass spectrometry Assay

The generation of M_1_dG in treated calf-thymus DNA was analyzed by mass spectrometry (Voyager DE STR from Applied Biosystems, Framingham, MA, USA), as previously reported [21]. DNA adducts were detected in the treated calf-thymus DNA through the following sequence of steps: (a) react DNA with NaBH_4_ (Merck, Kenilworth, NJ, USA) followed by precipitation with isopropanol (Merck, Kenilworth, NJ, USA); (b) digest with snake venom phosphodiesterase (Sigma, St Louis, MO, USA) and nuclease P1 (Sigma, St Louis, MO, USA); (c) extract DNA adducts that are less polar than normal nucleotides (nn) on an OASIS cartridge (Waters Corporation, Milford, MA, USA); (d) tag with an isotopologue pair of benzoylhistamines (d_o_ and d_4_) in a phosphate-specific labeling reaction in the presence of carbodiimide; (e) remove residual reagents by ion exchange solid-phase extraction; (f) resolve tagged adducts by capillary reversed-phase HPLC with collection of drops onto a MALDI plate; (g) add matrix (α-cyano-4-hydroxycinnamic acid); and (h) analyze by matrix-assisted laser desorption/ionization time-of-flight mass spectrometry.

### 4.6. ^32^P-Postlabeling Assay

M_1_dG generation was examined by post-labeling [16]. Briefly, DNA (2 μg) was incubated with micrococcal nuclease (21.4 mU/μL, Sigma, St Louis, MO, USA) and spleen phosphodiesterase (6.0 mU/μL, Sigma, St Louis, MO, USA) in hydrolysis buffer, pH 6.0 at 37 °C, for 4.5 h. Digested samples were treated with nuclease P1 (0.1 U/μL, Sigma, St Louis, MO, USA) at 37 °C for 30 min. Adducts were incubated with 7–25 μCi of carrier-free [γ-^32^P]ATP (3000 Ci/mM, GE Health Care, Amersham, UK) and polynucleotide kinase T_4_ (0.75 U/μL, Sigma, St Louis, MO, USA) to produce ^32^P-labeled DNA adducts in bicine buffer, pH 9.0, at 37 °C for 30 min. M_1_dG was analyzed by the following chromatography system: 0.35 M MgCl_2_ (Merck, Kenilworth, NJ, USA); 2.1 M lithium formate (Merck, Kenilworth, NJ, USA), 3.75 M urea (Merck, Kenilworth, NJ, USA) pH 3.75; and 0.24 M sodium phosphate (Merck, Kenilworth, NJ, USA), 2.4 M urea pH 6.4. DNA damage detection was done by storage phosphor imaging and intensifying screens (GE Health Care, Amersham, UK), that were scanned with Typhoon 9210 (GE Health Care, Amersham, UK). Adduct quantification was done by ImageQuant (GE Health Care, Amersham, UK). M_1_dG was expressed as relative adduct labeling (RAL × 10^8^) = pixel in adducted nucleotides/pixel in normal nucleotides, after background subtraction. Adduct levels were corrected across chromatographic experiments based on the recovery of the internal reference adduct standard.

### 4.7. Statistical Analysis

Data were log-transformed before statistical analysis. Group difference in HepG2 cells was examined by the *U* test of Mann–Whitney. Multivariate statistical analyses using log-normal regression models, including gender and age (continuous), as predictive variables, were conducted to analyze the relationship of oxidative nucleotide lesions with HCV-HCC. Mean Ratios (MR) estimates and its 95% Confidence Interval (C.I.) were used to measurement of effect for each level of predictor variables as compared to controls. Statistical analysis was done by SPSS 20.0 (SPSS, Chicago, IL, USA).

## Figures and Tables

**Table 1:** Levels of 7-hydro-8-oxo-2′-deoxyguanosine (8-oxodG) and 5-hydroxycytosine (5-OHC) at the codon 176 along the exon 5 sequence of the p53 gene among hepatitis C virus (HCV)-associated hepatocellular carcinoma (HCC) patients and controls, as well as hepatocytes in vitro treated with 0.2 mM xanthine plus 5.0 mU xanthine oxidase (XO)-treated. Frequency of 3-(2-deoxy-β-d-erythro-pentafuranosyl)pyrimido[1,2-α]purin-10(3H)-one deoxyguanosine (M_1_dG) are reported.

Oxidative Base Lesions at the Codon 176 of the p53 Gene and Generation of M_1_dG in Genomic DNA					
			**8-oxodG (–TGC–) ^a^**	**5-OHC (–TGC–) ^a^**	**M_1_dG**
		***N***	**RI ^b^ ± SE**	**RI ^b^ ± SE**	**RAL ^c^ × 10^8^ ± SE**
Hospital Based Population					
	Controls	22	0.28 ± 0.06	0.29 ± 0.01	1.67 ± 0.18
	HCV-HCC patients	31	0.61 ± 0.40	0.95 ± 0.27	3.25 ± 0.41
Experimental Hepatocytes					
	Untreated HepG2 cells	6	0.20 ± 0.01	0.22 ± 0.02	0.35 ± 0.2
	XO-treated HepG2 cells	6	0.34 ± 0.03	0.44 ± 0.04	3.2 ± 0.3

^a^ Underlined letters indicate the residues for which base lesions are reported; ^b^ Relative Intensity (RI) ± Standard Error (SE); ^c^ Relative adduct labeling (RAL) × 10^8^ normal nucleotides ± SE.

**Table 2:** Mean Ratio (MR) and 95% Confidence Interval (C.I.) of 7-hydro-8-oxo-2′-deoxyguanosine (8-oxodG) and 5-hydroxycytosine (5-OHC) at the codon 176 along the exon 5 sequence of the p53 gene among hepatitis C virus (HCV)-associated hepatocellular carcinoma (HCC) patients and controls. MR estimates of 3-(2-deoxy-β-d-erythro-pentafuranosyl)pyrimido[1,2-α]purin-10(3H)-one deoxyguanosine (M_1_dG) are shown.

Mean Ratio and 95% Confidence Interval of Biomarkers Under Study			
	8-oxodG (–TGC–) ^a^	5-OHC (–TGC–) ^a^	M_1_dG
	MR, 95% C.I.	MR, 95% C.I.	MR, 95% C.I.
Controls	Reference	Reference	Reference
HCV-HCC patients	2.3, 1.57–3.48	3.8, 0.71–20.3	1.87, 1.27–2.77
*p*-value ^b^	<0.0001	0.115	0.001

^a^ Underlined letters indicate the residues for which lesions are reported; ^b^ *p*-values referred to comparison with the reference level after correction for age and gender.

**Table 3:** Relationship between the levels of 7-hydro-8-oxo-2′-deoxyguanosine (8-oxodG) and 5-hydroxycytosine (5-OHC) at the codon 176 along the exon 5 sequence of the p53 gene among hepatitis C virus (HCV)-associated hepatocellular carcinoma (HCC) patients and the spectrum of transition and transversion p53 mutations in HCV-associated HCC tumors. Mean Ratio (MR) and 95% Confidence Interval (C.I.) are shown.

Position ^a^	Nomenclature	DNA Adducts	RI ± SE ^b^	Mean Ratio, 95% C.I.	Mutation Type	Mutation Rate
(–TGC–)	c.527G	8-oxodG	0.61 ± 0.40	130%, 95% C.I. 1.57–3.48	G:C > A:T	41.67%
(–TGC–)	c.528C	5-OHC	0.95 ± 0.27	280%, 95% C.I. 0.71–20.3	G:C > T:A	8.33%

^a^ Underlined letters indicate the residues indicate the residues for which base lesions are reported; ^b^ Relative intensity (RI) ± Standard Error (SE).
